# MU2 and HP1a Regulate the Recognition of Double Strand Breaks in *Drosophila melanogaster*

**DOI:** 10.1371/journal.pone.0025439

**Published:** 2011-09-23

**Authors:** Raghuvar Dronamraju, James M. Mason

**Affiliations:** Laboratory of Molecular Genetics, National Institute of Environmental Health Sciences, Research Triangle Park, North Carolina, United States of America; Duke University, United States of America

## Abstract

Chromatin structure regulates the dynamics of the recognition and repair of DNA double strand breaks; open chromatin enhances the recruitment of DNA damage response factors, while compact chromatin is refractory to the assembly of radiation-induced repair foci. MU2, an orthologue of human MDC1, a scaffold for ionizing radiation-induced repair foci, is a widely distributed chromosomal protein in *Drosophila melanogaster* that moves to DNA repair foci after irradiation. Here we show using yeast 2 hybrid screens and co-immunoprecipitation that MU2 binds the chromoshadow domain of the heterochromatin protein HP1 in untreated cells. We asked what role HP1 plays in the formation of repair foci and cell cycle control in response to DNA damage. After irradiation repair foci form in heterochromatin but are shunted to the edge of heterochromatic regions an HP1-dependent manner, suggesting compartmentalized repair. Hydroxyurea-induced repair foci that form at collapsed replication forks, however, remain in the heterochromatic compartment. HP1a depletion in irradiated imaginal disc cells increases apoptosis and disrupts G2/M arrest. Further, cells irradiated in mitosis produced more and brighter repair foci than to cells irradiated during interphase. Thus, the interplay between MU2 and HP1a is dynamic and may be different in euchromatin and heterochromatin during DNA break recognition and repair.

## Introduction

Homologous recombination and nonhomologous end joining are the two major mechanisms for repair of double strand breaks (DSBs) in DNA, ensuring the transmission of intact genetic information. While regulated generation of DSBs by cellular enzymes is an essential event during meiosis [1] and VDJ recombination [2], DSBs produced by environmental stimuli are mostly deleterious if left unrepaired [3]. DSBs induce cellular signals, which are primarily dependent upon the activation of the ATM kinase and culminate in the recruitment of DNA damage response (DDR) proteins to the break. Phosphorylation of H2AX (*Drosophila* homolog: H2Av) to produce γH2AX is one of the earliest chromatin modifications that sets the stage for the assembly of multi-protein complexes that are microscopically discernible as foci [4], [5], [6]. Processing of DSBs is different in heterochromatin and euchromatin, as evidenced by the preferential formation of γH2AX foci in euchromatin [7].

We have described an ionizing radiation-dependent mutator (*mu2*) of *Drosophila* that increases the recovery of terminal deficiencies, i. e. chromosomes that have lost a telomere [8], [9], [10], [11]. Extensive genetic analysis suggested that MU2 may be a chromatin protein and play an important role in the repair of radiation-induced DSBs [10]. MU2 protein primarily localizes to the oocyte nucleus during meiotic recombination. The polytene chromosomes are covered with MU2 in a pattern similar to DAPI staining. A striking feature of the protein is the presence of a C-terminal tandem BRCA1 C-terminal (BRCT) domain, a phospho-protein binding domain, which is a feature of many proteins known to be involved in DNA repair and cell cycle control. An N-terminal forkhead associated domain has also been identified. Based on amino acid sequence, domain architecture, protein interactions, and cellular localization, MU2 appears to be an orthologue of human MDC1 [12].

Heterochromatin protein 1a (HP1a) was originally discovered in *Drosophila* by virtue of its localization to the DAPI-rich, heterochromatic regions on polytene chromosomes [13]. HP1 homologues exist in almost all eukaryotes and are well conserved [14]. HP1a in *Drosophila* is a 206 amino acid polypeptide that functions in heterochromatic gene silencing [15], [16], [17], transcription regulation [18], telomere capping, and position effect of variegation [19], [20]. The *Drosophila* genome encodes five HP1 paralogues, HP1a-e, as compared with three vertebrate paralogues α, β and γ. *Drosophila* HP1 paralogues are different from each other and may not be orthologous to any of the vertebrate paralogues [21]. The role of HP1 paralogues in DNA damage recognition and repair is not known in *Drosophila* and is matter of debate in vertebrates. Heterochromatin formation requires histone modifications, such as trimethylation of histone H3 at Lys 9 to produce H3K9Me3, which is directly involved in the recruitment of HP1a to specific regions of the genome, suggesting that HP1a is important for this process [22], [23].

Yeast 2 hybrid results showed that MU2 interacts with HP1a, suggesting a role for HP1a in DNA damage recognition. In light of the emerging role of vertebrate paralogues of HP1 in DNA damage response [24], we initiated studies to understand the role of *Drosophila* HP1a in the recognition of DSBs. We show that HP1a, -b and -c are not recruited to ionizing radiation (IR) induced foci (IRIFs) or laser induced breaks. γH2Av and MU2 foci co-localize in HP1a-rich heterochromatic regions upon treatment with hydroxyurea (HU) but only weakly after irradiation. Interestingly, IRIFs that are formed in heterochromatin tend to migrate and accumulate at the periphery of the chromocenter, an effect not observed upon HU treatment. Depletion of HP1a not only prevented this effect but also caused defects in mitotic progression. Further, cells irradiated during mitosis, during which HP1a is depleted from chromosomes, accumulated γH2Av foci, but the foci disappeared at telophase. These results suggest an underlying role of chromatin configuration in the recognition of DSBs that is regulated by the interaction of chromatin proteins.

## Results

### Interaction of HP1 and MU2

**Figure 1:**
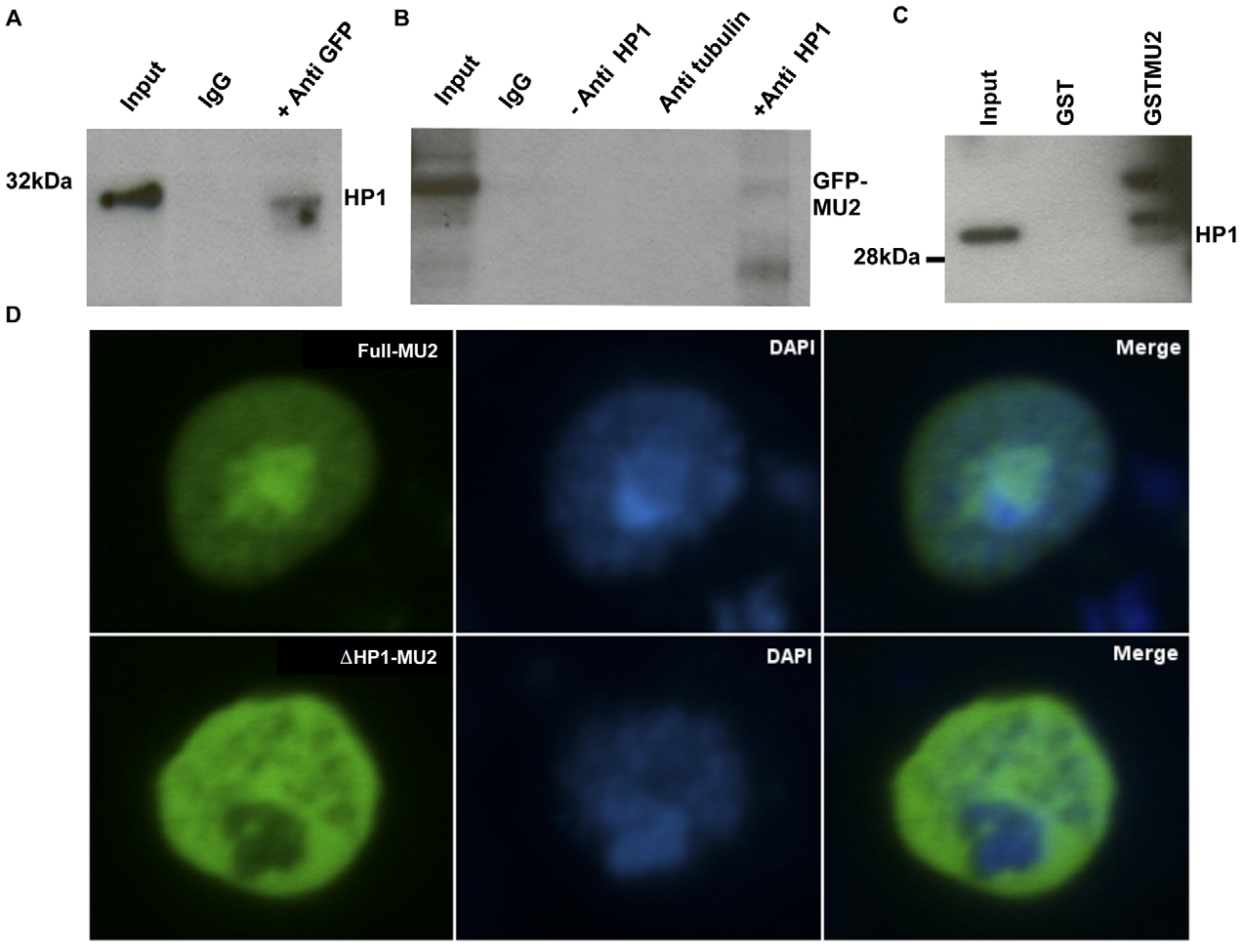
Interaction of MU2 and HP1a. Nuclear extracts were prepared from mGFP-MU2 embryos, and pulldowns were performed using anti-GFP and anti-HP1a antibodies. (A) Western blots were performed to detect HP1a in the immunoprecipitates of mGFP-MU2 from Drosophila embryos. (B) mGFP-MU2 was detected from immunoprecipitation reactions performed using anti-HP1a antibodies from transgenic mGFP-MU2 embryos. (C) The interaction between HP1a and MU2 was detected *in vitro* in GST pull down experiments using a GST-MU2 fragment as bait and nuclear extracts prepared from S2 cells as prey. A fragment of MU2 (aa 900–1000) was cloned in the pGEX4T1 vector and immobilized on glutathione sepharose beads. Beads were incubated with nuclear extracts from S2 cells, washed extensively and detected by western with anti-HP1a antibodies. (D) S2 cells were transfected with the vectors encoding full length MU2 or truncated MU2 as eGFP tagged constructs, as described in materials and methods. Co-localization of eGFP tagged full length MU2 protein (Full-MU2) in the DAPI-rich region is seen in cultured S2 cells (upper row of panels), the MU2 protein deleted of the HP1a binding region (ΔHP1-MU2) is excluded from DAPI-rich regions (lower row of panels). DAPI rich regions corresponding to the HP1a-rich regions.

Yeast 2 hybrid (Y2H) analysis was conducted by Myriad Biotech, USA [25], using fragments of MU2 as bait and a 0–12 h embryonic cDNA or an S2 cell library as prey (Fig. S1). Four fragments that included the region of MU2 preceding the tandem BRCT domain (aa 900–1000) showed a large number of hits, but no hits were found with other fragments, suggesting a possible interaction between MU2 and HP1a in this domain. These results also indicate that this region of MU2 interacts with the chromoshadow domain of HP1a, which is a well-established domain for protein-protein interactions. To confirm the Y2H results, we performed a co-immunoprecipitation of MU2 and HP1a. Since the MU2 antibodies we generated do not work for western blots, we used the transgenic mGFP-MU2 flies [12] that express MU2 protein using its own promoter [26] and performed co-immunoprecipitations with anti-GFP and anti-HP1a polyclonal antibodies. HP1a is co-immunoprecipitated using anti-GFP antibodies and vice versa (Fig. 1).

To confirm the binding of the internal region (aa 900–1000) of MU2 to HP1a, we performed GST pulldown experiments using a GST-MU2 fragment as bait and nuclear extracts prepared from S2 cells as prey. Western analysis using anti-HP1a antibodies confirmed the presence of HP1a in the GST-MU2 lane but not the GST-only lane, suggesting that this region indeed binds to HP1a (Fig. 1C). HP1a is a major component of pericentric heterochromatin [13] and co-localizes with the DAPI-rich chromocenter in diploid cells [27]. To understand the nature of the interaction between MU2 and HP1a, we transfected S2 cells with vectors encoding full length MU2 and a MU2 construct deleted of the HP1a binding region and expressed them as eGFP fusion proteins. Full length MU2 protein co-localized with both the DAPI-rich and the DAPI-poor regions, whereas the MU2 construct deleted of the HP1a binding region localized only to the DAPI-poor regions (Fig. 1D).

At cycle 14 of embryogenesis the dividing nuclei start migrating to the peripheral region forming the blastoderm. Pericentric heterochromatin is known to occupy a distinct location in these nuclei after migration [28]. Immunostaining experiments were performed on cycle-14 embryos to ask whether MU2 and HP1a interact during this stage of development. There was no co-localization of mGFP-MU2 and HP1a in cycle-14 embryos, as mGFP-MU2 is primarily in the yolk, while HP1a is primarily nuclear (Fig. S2). To understand if mutations in *mu2* have an effect on the localization of HP1a, we immunostained wild type and *mu2^a^* cycle-14 embryos. Fig. S2 demonstrates that in the mutant embryos there is no defect in the localization of HP1a to nuclei at this stage of embryogenesis, although migration of nuclei to the blastoderm surface may be delayed. Further, we stained *mu2^a^* mutant polytene chromosomes with anti-HP1a antibodies and did not observe any significant changes in the localization of HP1a, suggesting that MU2 is not required for proper localization of HP1a on polytene chromosomes.

### Dynamics of γH2Av foci in heterochromatin

**Figure 2:**
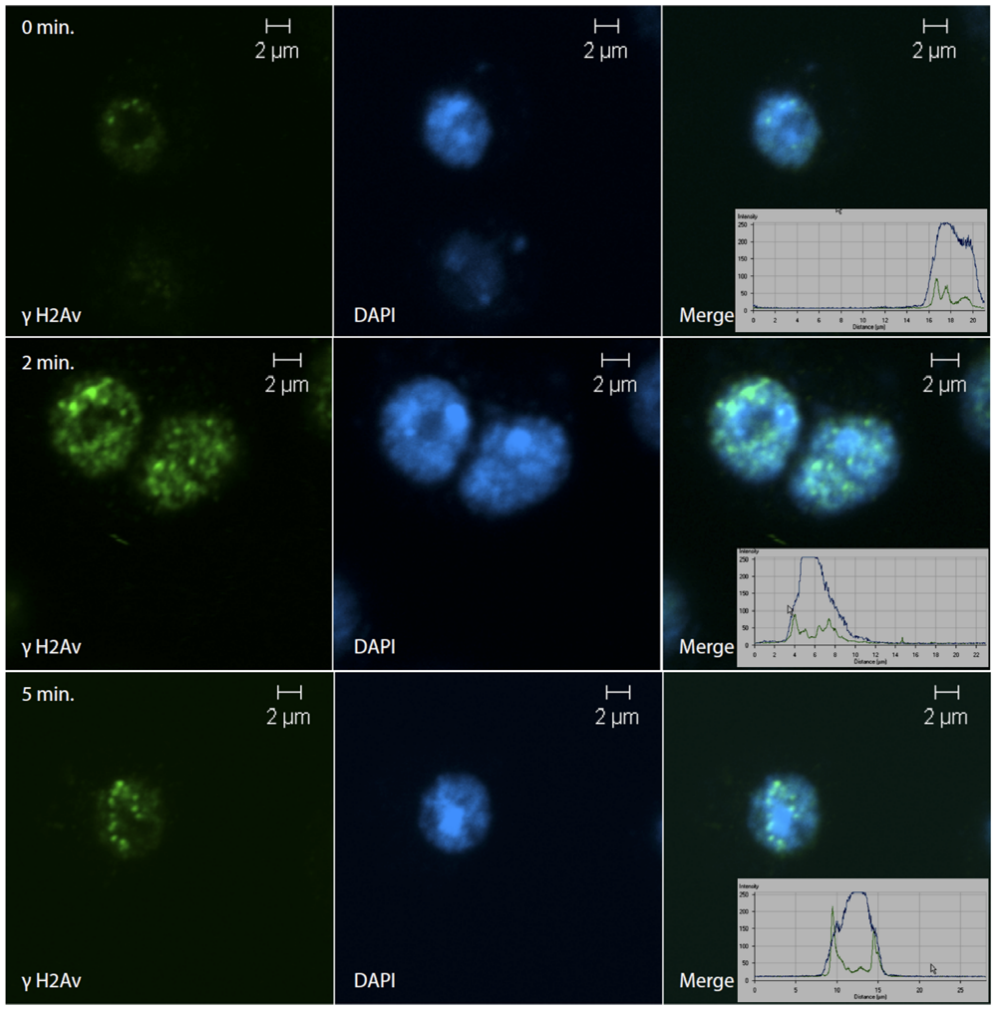
Expulsion of γH2Av foci from heterochromatin. S2 cells were cultured under logarithmic conditions, plated into 8-well chambered plates and exposed to irradiation. Cells received a dose of 25 Gy in 30 seconds and fixed immediately, or 2 or 5 min after irradiation. At 0 min a few small γH2Av foci are found in DAPI-rich regions (first row). After two minutes these grow and show a definite migration (second row). By five minutes the foci are at the periphery of the heterochromatic regions (third row). γH2Av is shown in green. Insets are the fluorescence profiles on γH2Av (green) and DAPI (blue).

The formation of IRIFs differs in heterochromatin and euchromatin with the formation being preferential to euchromatin [7], [29]. In our initial experiments using 25 gray (Gy) delivered at 5 Gy/min, repair foci were found at the periphery of heterochromatin, which has also been observed by Goodarzi *et al.* [30]. When we decreased the time of irradiation by using a higher dose rate (50 Gy/min) and fixed cells after 0, 2 or 5 minutes, γH2Av foci were formed in DAPI-rich regions immediately after irradiation, but within 5 minutes most of them were at the periphery of the DAPI-rich region (Fig. 2), suggesting the migration of IRIFs away from the chromocenter. The number and the intensity of foci was low immediately after treatment but increased with time. Of the 100 interphase cells analyzed at each time point, the number of cells showing migration of foci was almost 100% in three independent experiments. The only cells that did not show migration of foci were in mitosis and could be distinguished by their larger size.

**Figure 3:**
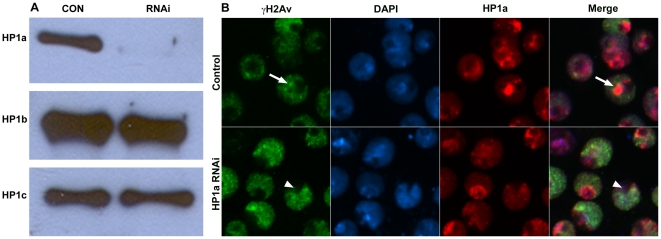
Effects of HP1a depletion on focal movement. S2 cells were treated with dsRNA specific to HP1a for 3 days. (A) At the end of treatment cells were harvested and used for westerns to detect the change in protein levels or plated for irradiation. The western blot shows a decrease in the level of HP1a, but not HP1b or -c. (B) The effects of HP1a dsRNA on the migration of the γH2Av foci to the periphery of heterochromatin are shown. Cells were treated to 25 Gy (5 Gy/min), fixed using 4% paraformaldehyde and stained with anti-γH2Av (green), anti-HP1a (red), and DAPI (blue). In control cells (upper row) the foci are at the periphery of the DAPI-rich regions (arrow). In HP1a dsRNA treated cells (lower row) the DAPI-rich regions are not well organized, and the repair foci are not expelled from these regions. The extreme DAPI-poor regions (arrowhead) may be nucleoli and do not accumulate repair foci.

To ask whether the migration of IRIFs depends on the level of HP1a protein, we performed RNAi knockdown of HP1a in S2 cells irradiated at 50 Gy/min. Treatment of S2 cells with a HP1a double stranded RNA (dsRNA) generated from the full-length cDNA reduced the level of HP1a protein significantly without affecting the levels of HP1b or -c (Fig. 3A). The intensely HP1a-stained chromocenter is not seen in the treated cells; rather, there is a weak HP1a stain throughout most of the nucleus. Many nuclei contain a DAPI-poor, HP1a-poor region, which is likely the nucleolus (Fig. 3, arrowhead). Immunocytochemistry using anti-γH2Av showed that, in control S2 cells 5 min after irradiation, the repair foci were on the chromocenter periphery, as observed previously (Fig. 3B, arrow), whereas in HP1a-depleted cells the foci are distributed over the nucleus with no differentiation between euchromatin and heterochromatin (Fig. 3B), with the exception that the presumptive nucleolus does not accumulate foci. Some cells in the dsRNA-treated population are not transfected and still exhibit a clear HP1a-rich chromocenter that excludes repair foci. One of these is shown to the left of the arrowhead in Fig. 3B.

### HP1 Proteins are not recruited to DNA damage foci

**Figure 4:**
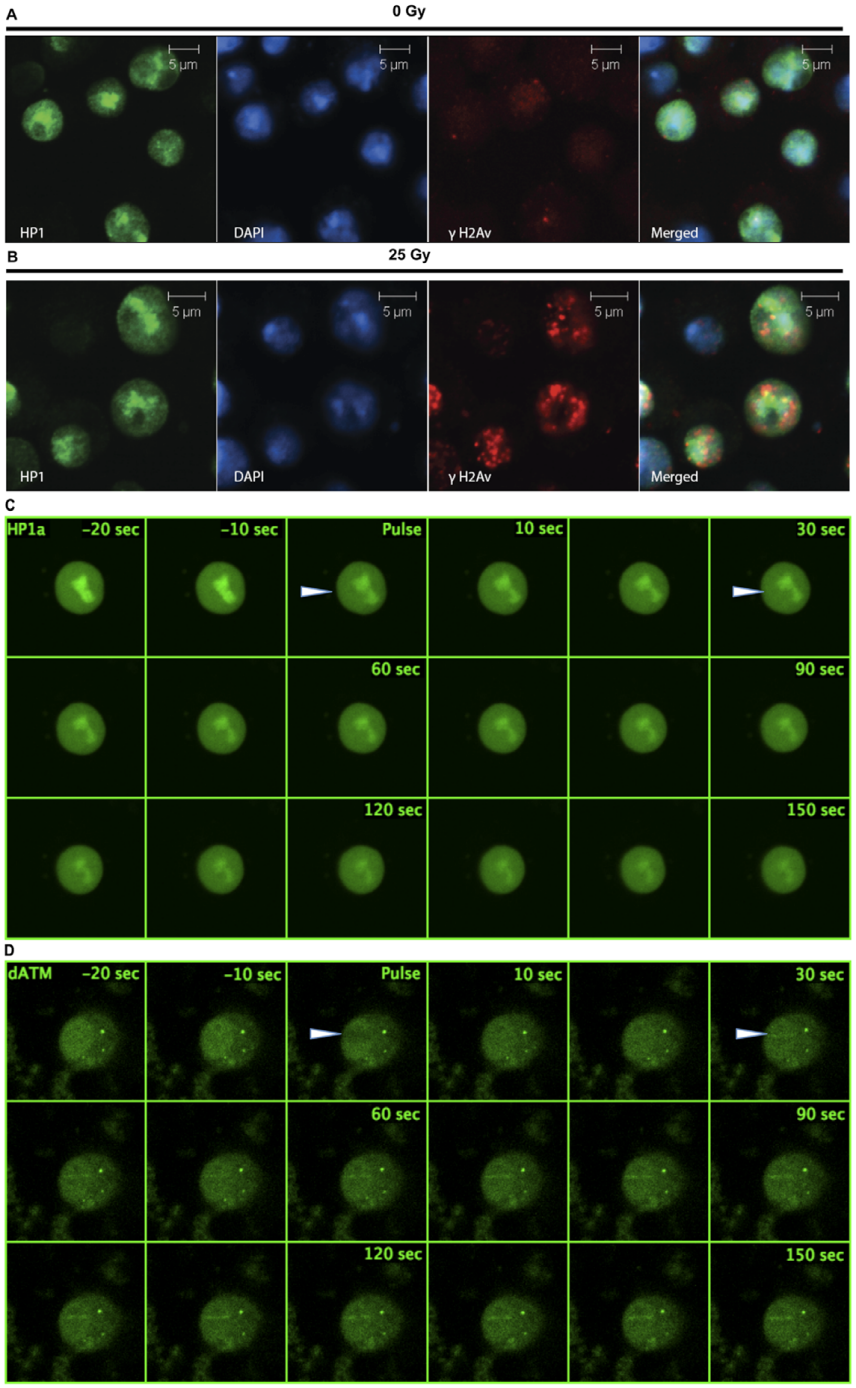
HP1a is not recruited to IRIF and laser induced breaks. Cultured S2 cells were irradiated with 25 Gy (5 Gy/min), fixed using 4% paraformaldehyde and immunostained using anti-γH2Av antibodies (red), as a mark for DSBs and anti-HP1a antibodies (green). DNA was identified by DAPI (blue). (A) Unirradiated S2 cells immunostained with HP1a and γH2Av antibodies show localization of HP1a to DAPI-rich regions and minimal staining of γH2Av. (B) The HP1a staining pattern relative to DAPI in irradiated cells does not change in comparison to controls, and HP1a does not co-localize with the γH2Av foci upon irradiation. Note that the γH2Av foci are almost always on the periphery of the DAPI-rich regions. (C) Dynamics of HP1a proteins in cells transfected with eGFP-HP1a, treated with a 364 nm laser in a region designated by the arrowhead and followed for 150 sec. HP1a is not recruited to the laser induced breaks, although the overall level of HP1 in the nucleus, especially in the chromocenter, seems to decrease. (D) Dynamics of ATM in cells transfected with EGFP-ATM and treated with a 364 nm laser in a region designated by the arrowhead and followed for 150 sec. ATM can be seen in the treated region by 30 sec and remains for at least another two minutes.

Given the findings that γH2Av foci migrate from heterochromatin and the recent reports that HP1 homologues are recruited to DSBs in mammalian cells [31], [32], we asked whether HP1a is recruited to IRIFs. We irradiated S2 cells, fixed them and stained with anti-γH2Av and anti-HP1a antibodies. As can be seen in Fig. 4, unirradiated cells show minimal γH2Av staining, and the HP1a protein is primarily localized to the DAPI-rich regions. Upon irradiation at a dose of 25 Gy (5 Gy/min) γH2Av foci are formed in a robust manner, whereas there is no discernible change in the localization of HP1a protein in irradiated cells. The γH2Av foci are mostly located at the periphery of the HP1-rich regions, suggesting an expulsion from the heterochromatin. HP1b and HP1c are also not found at the sites of radiation induced foci under these conditions (Fig. S3).

Since our studies on the recruitment of HP1 paralogues were done using cells fixed after irradiation, it is possible that the time between irradiation and fixing may interfere with the interpretation of our results. We therefore performed real time imaging of the DSBs generated using laser scanning confocal microscopy. Cells were transfected with eGFP-tagged HP1a or ATM constructs, sensitized with 10 µM BrdU and irradiated with 364 nm continuous wave laser. ATM undergoes a series of posttranslational modifications and is recruited to the DSBs [33]. eGFP-ATM is recruited to the sites of laser induced DSBs within 30 seconds of irradiation (Fig. 4D) in a dose depended manner. HP1a is not recruited to the sites of the laser induced DSBs, rather there was a decrease in the fluorescence with time, suggesting that HP1a is ejected from the sites of DSB or possibly from the chromocenter as a whole (Fig. 4D). To confirm the accumulation of DDR proteins at laser-induced breaks we performed studies using eGFP-tagged H2Av in S2 cells. As shown in Fig. S4, H2Av localized robustly to the treated area.

### Collapsed replication forks in heterochromatin

**Figure 5:**
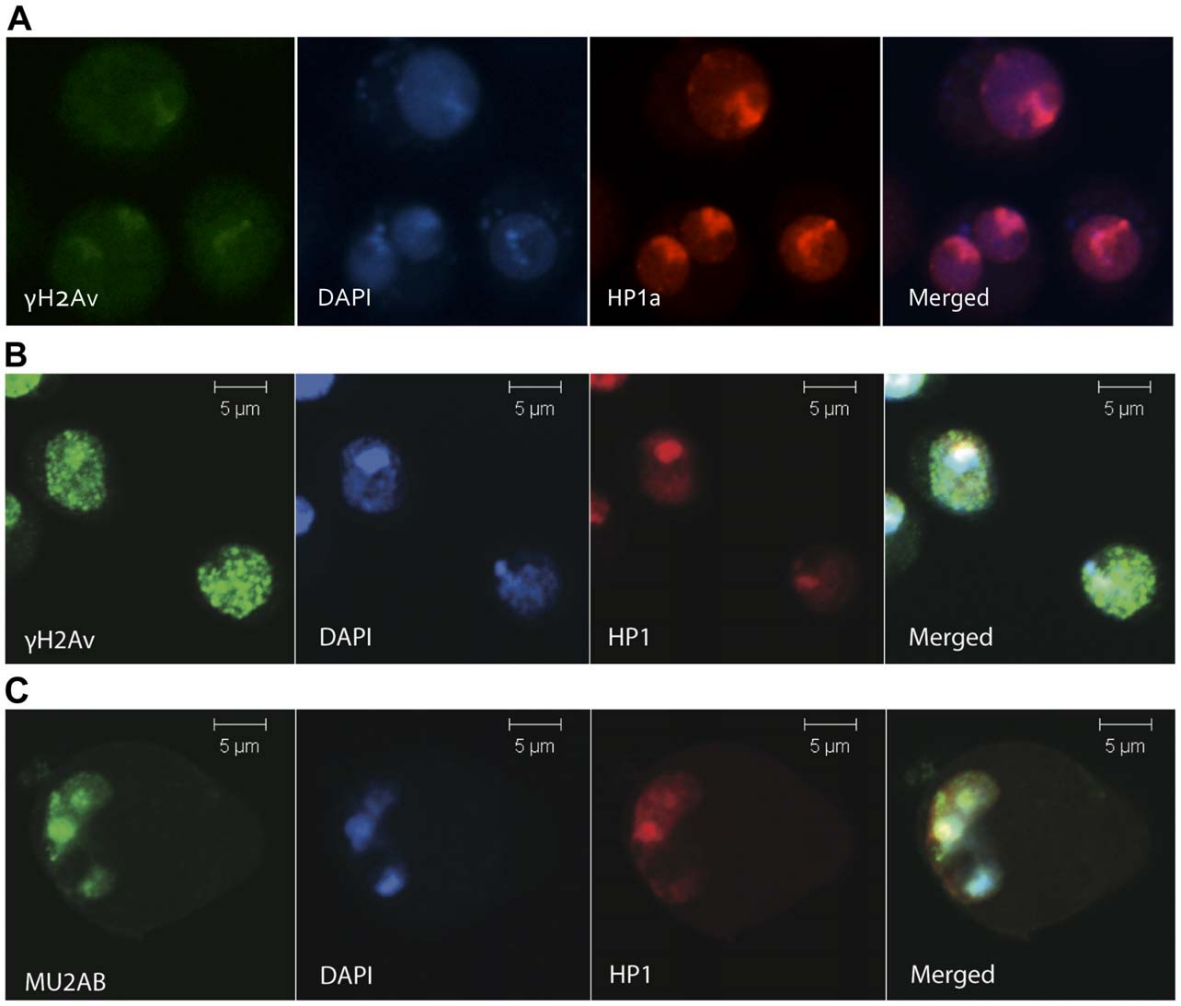
MU2 and γH2Av recognize stalled replication forks. Experiments were conducted to understand the interaction between HP1a and MU2 in heterochromatin. Cells were treated with HU for 16 h, fixed in 4% paraformaldehyde and observed using confocal microscopy. Staining of S2 cells using anti-γH2Av or anti-MU2 (green) as a mark for DSBs, anti-HP1a (red), and DAPI (blue). (A) Control S2 cells treated with PBS. (B) γH2Av foci are formed in the DAPI rich regions and co-localize with HP1a protein. Note that the foci formed upon HU treatment are not on the periphery of the DAPI-rich domain. (C) MU2 protein co-localized with the HP1a in HU treated cells.

It is clear from the above results that heterochromatin and the associated proteins have an effect on the dynamics of γH2Av foci. We therefore asked whether there are differences between radiation induced breaks and those induced by collapsed replication forks. Locally under-replicated regions in the polytene chromosomes of *Drosophila melanogaster* are known to accumulate γH2Av, suggesting that local under-replication activates the DDR [34]. We induced damage in cultured S2 cells by treating with HU, which depletes deoxyribonucleotide triphosphate pools, stalls replication forks, arrests cells in S phase, and induces genomic instability [35]. Untreated cells show few repair foci (Fig. 5A). Treatment of S2 cells with 10 mM HU for 16 h induced an S-phase arrest. We stained the HU-treated cells with anti-γH2Av and anti-HP1 monoclonal antibodies and observed that γH2Av foci are formed in the HP1a-rich, DAPI-rich nuclear regions, (Fig. 5B). MU2 foci also form in the HP1a rich regions (Fig. 5B). The migration that we observed for the foci upon irradiation was absent after HU treatment, suggesting an intrinsic difference in the nature of the damage.

### Dynamics of HP1a and γH2Av foci in mitosis

**Figure 6:**
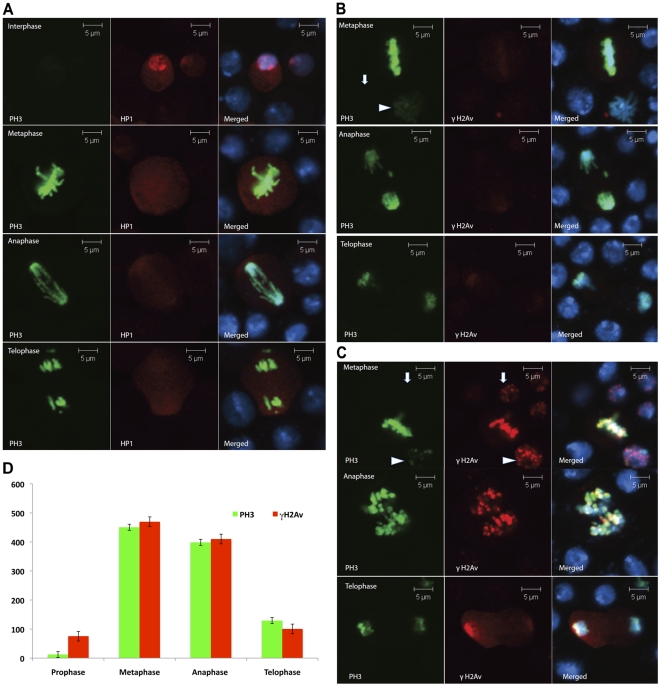
HP1 and γH2Av dynamics during mitosis. Non-synchronized S2 cells were grown under logarithmic conditions, plated in 8-well chambered slides and exposed to irradiation of 25 Gy (5 Gy/min). Immediately after irradiation S2 cells were immunostained and scored for mitotic cells under a confocal microscope based on PH3 staining. (A) Cells were fixed and stained with anti-HP1a antibody (red) and anti-PH3 (green). Different phases of mitosis are shown. (B) Unirradiated cells stained with anti-γH2Av (red) and anti-PH3 (green) showing an absence of IRIFs. (C) γH2Av foci are formed in irradiated mitotic cells. Staining as in (B). An interphase cell (arrow) and a cell in late prophase (arrowhead) are shown in the upper row. The middle and lower rows show cells in anaphase and telophase. Note that the γH2Av foci are less intense at telophase. (D) Graph showing the intensity of chromosomal γH2Av staining at various stages of mitosis relative to PH3, a marker for mitotic chromosomes.

The chromodomain of HP1a binds to the histone mark, H3K9Me3, and performs the important function of heterochromatin maintenance. The same histone H3 when phosphorylated at Ser 10 (PH3) ejects HP1 proteins (all paralogues) from their binding site and helps in mitotic chromosome condensation [14]. We exploited this situation to study the effects of loss of HP1a from chromosomes on the formation of γH2Av foci. Immediately after irradiation with 25 Gy at 5 Gy/min cells were stained with anti-PH3 to identify mitotic chromosomes and anti-γH2Av to identify IRIFs. Interphase S2 cells show a clear localization of HP1a at the chromocenter. At metaphase, anaphase and telophase, HP1a staining is primarily nonchromosomal (Fig. 6A), although in mitotic spreads some HP1 staining can be seen at specific sites on the chromosomes [36]. Unirradiated mitotic cells do not show immunostaining for γH2Av (Fig. 6B). Irradiation of mitotic cells produced γH2Av foci that were brighter than the γH2Av foci in interphase cells. The intensity of γH2Av foci was similar in metaphase and anaphase, but as the cells entered telophase there was a decrease in the γH2Av staining (Fig. 6C). If the epitope accessibility of γH2Av is lost during chromosome condensation, we would expect a loss of signal for γH2Av, not an increase in signal. To control for epitope accessibility, we made a comparison to PH3 (Fig. 6D) and show that the signal for γH2Av increases in mitotic chromosomes in concert with the mitosis-specific marker. Cells that have initiated the process of chromosome decondensation at telophase and show intermediate staining for PH3 also exhibit a decrease in the intensity of γH2Av foci (Fig. 6C). Thus, it seems that the intensity of γH2Av foci is particularly strong on mitotic chromosomes that are naturally depleted for HP1a.

### Radio-resistance of HP1a depleted S2 cells

**Figure 7:**
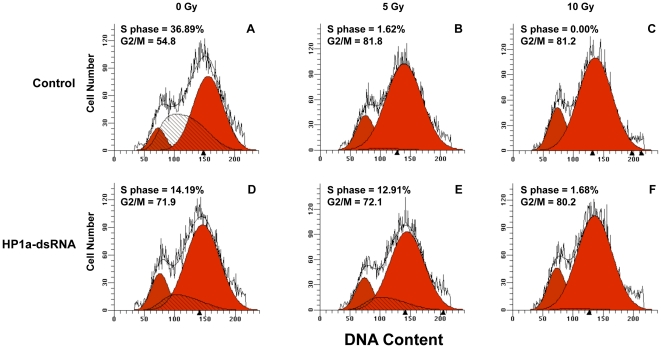
HP1a depletion affects cell cycle in S2 cells. Control or HP1a dsRNA-treated S2 cells were irradiated and stained with propidium iodide and subjected to FACS analysis. Control cells that were unirradiated (A), treated with 5 Gy (B) or 10 Gy (C) of ionizing radiation are shown in the top row. HP1a-dsRNA treated cells with 0 Gy (D), 5 Gy (E) and 10 Gy (F) of IR are shown in the bottom row. G1-phase cells are shown in the red peak to the left, G2/M cells are shown in the red peak to the right, and S-phase cells are shown in the hatched peak. Mitotic cells comprise 3–4% of an asynchronous population of S2 cells. Therefore, most of the cells in the G2/M peak are in G2. Proportions of cells at S and G2/M phases of the cycle are also shown numerically.

Cells in S phase are known to be relatively radiosensitive in comparison to cells in the other phases of cell cycle [37]. Treatment of S2 cells with HP1a dsRNA leads to a decrease in the number of cells in S phase and an apparent increase in the number of cells at both G1 and G2/M (Fig. 7). The decrease in S phase cells is accompanied by an increase in apoptotic cells [18]. To ask whether HP1a is involved in modulating the response to IR, we irradiated control and HP1a-dsRNA treated cells at 5 and 10 Gy and immediately analyzed cell cycle parameters using FACS analysis of propidium iodide stained nuclei. After 5 Gy of IR the control shows an almost complete lack of S phase cells, as might be expected if these cells are hypersensitive to radiation damage. The HP1a depleted S phase cells, on the other hand, are resistant to killing by 5 Gy of IR. After 10 Gy, however, HP1 depleted S phase cells are also dead. This suggests that HP1a plays a role in radiation sensitivity during DNA replication, either directly or indirectly.

### HP1a regulates G2/M checkpoint in wing imaginal discs

**Figure 8:**
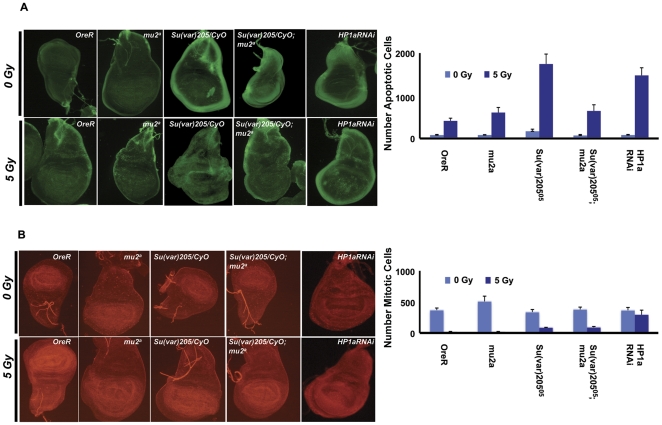
Apoptosis and G2/M checkpoint in wing discs. Apoptosis and the regulation of mitotic checkpoints in the wing imaginal discs are shown in response to IR. Wing imaginal discs from OreR (control), homozygous *mu2^a^*, heterozygous *Su(var)205^05^/CyO*, and double mutant *Su(var)205^05^/CyO; mu2^a^* third instar wandering larvae were dissected 3 h after ionizing irradiation and stained with acridine orange. Briefly, the discs were dissected in PBS, rinsed twice with PBS, mounted and observed under a fluorescence microscope. (A) The discs were incubated in acridine orange, which detects apoptosis as green spots in the body of the discs. The data are graphed as the mean + standard deviation of counts from four discs each in three independent experiments. (B) Discs were fixed in 4% paraformaldehyde, block permeabilized and immunostained with anti-PH3 antibody to detect mitosis. The data are graphed as the mean + standard deviation of counts from four discs each in three independent experiments.

Imaginal discs of Drosophila third instar larvae are an ideal system to study the effects of gene mutations on cell cycle regulation [38]. We exploited this system to understand the role played by mutations in the *Su(var)205* (which encodes HP1a) and *mu2* genes in regulating the cell cycle. We also used the GAL4-UAS system [39] to down-regulate the expression of HP1a. Mutations in *Su(var)205* are homozygous pupal lethal, and the wing imaginal discs of these homozygous animals show high levels of spontaneous apoptosis. Mutations in certain components of the DDR pathway are known to decrease the levels of spontaneous apoptosis. We irradiated wild type controls, heterozygous *Su(var)205^05^/CyO*, homozygous *mu2^a^* single mutants as well as *Su(var)205^05^/CyO*; *mu2^a^* double mutants, and stained wing imaginal discs with acridine orange three hours after irradiation to monitor apoptosis. There was an increase in the number of apoptotic cells in the *Su(var)205^05^/CyO*, the *mu2^a^*, and the *Su(var)205^05^/CyO*; *mu2^a^* discs compared to the controls (Fig. 8A). *Su(var)205^05^/CyO* imaginal discs showed a 4-fold increase in the number of apoptotic cells compared with wild type, and a comparable increase was seen in the HP1a RNAi discs. The number of apoptotic cells is significantly lower in the *Su(var)205^05^/CyO*; *mu2^a^* discs, suggesting that HP1a interacts with MU2 and is important for the repair of DSBs, or that the *mu2^a^* apoptotic response is epistatic to the *Su(var)205* response.

To understand the role of HP1a in the regulation of the G2/M checkpoint *in vivo*, wing imaginal discs were dissected from wandering third instar larvae three hours after mock irradiation or irradiation at a dose of 5 Gy. Discs were stained with the mitosis specific marker PH3 using anti-PH3 antibody, and the number of mitotic cells counted. While wild type and *mu2^a^* discs showed minimal numbers of mitotic cells after irradiation, there were a considerable number of mitotic cells in the *Su(var)205^05^/CyO* mutant discs and the HP1a depleted discs, suggesting that HP1a controls the G2/M checkpoint (Fig. 8B). The number of mitotic cells was higher in the RNAi flies compared to *Su(var)205^05^/CyO* mutants, which may be because the mutation is heterozygous, and therefore a stronger effect may be achieved by dsRNA treatment.

## Discussion

### Significance of HP1a and MU2 interaction

Recognition and repair of DNA damage is a complex process controlled by many factors whose interactions dictate the outcome. Here we show that MU2, a scaffold for IRIFs, interacts with HP1a. A region in MU2 adjacent to the C-terminal BRCT domain interacts with the chromoshadow domain of HP1a in the absence of radiation treatment. Ayoub *et al.* [31] and Lujisterburg *et al.* [32] have studied the role played by HP1 paralogues in DNA damage response in mouse cells. While the former group suggested that there is a local ejection of HP1β from the site of a DSB within seconds, the latter group proposed that the three mammalian HP1 paralogues (HP1α, -β and -γ) are recruited to different kinds of DNA damage over a longer time period. The proposal of a bimodal nature to HP1 dynamics at DNA damage sites, quickly leaving and slowly returning, solved this apparent discrepancy [40]. The exact status of HP1 is not known, given the fact that the three paralogues show different distribution, although the recruitment of HP1 paralogues to different kinds of damage was dependent on their chromoshadow domains [24]. We observed, using IR and laser induced DNA damage experiments, that three *Drosophila* HP1 paralogues are not recruited to the DSBs induced by irradiation.

In mammalian cells γH2AX foci are formed preferentially in euchromatin [7], [29], suggesting that the heterochromatin is less accessible to DDR components [41]. However, if DNA damage is induced during S phase using HU, γH2AX foci are formed in heterochromatin [7]. Similarly, we found that HU-induce foci are formed in heterochromatin and do not move to the periphery of the chromocenter. It is possible that the HU-induced repair foci remain in the chromocenter because this chromatin needs to be relaxed in order to be replicated, and foci in the temporarily relaxed chromatin do not have to find a more open region.

Since Drosophila HP1a is primarily localized to heterochromatin, we inspected the interaction between HP1a and MU2 in heterochromatin. In S2 cells we observed localization of MU2 in heterochromatin, which depended on its HP1a binding site. Y2H experiments suggested an interaction between these two proteins in early embryos, but MU2 primarily localizes to the blastoderm cytoplasm, rather than the nuclei. It is possible that any interaction at this stage is due to the formation of a multi-protein complex deposited in the embryo maternally, as HP1 is known to form such complexes [28].

The mechanisms of DNA break repair are different in heterochromatin and euchromatin, underlining the differences in chromatin organization. While ATM is dispensable for the repair of euchromatic breaks, it is an essential component for the repair of heterochromatic breaks [42]. Further, knockdown of important heterochromatic components, such as KAP1, obviates the requirement for ATM, suggesting that key components of heterochromatin provide some resistance to DNA damage recognition and repair and are modified by the ATM kinase to allow repair to occur [30], [43], [44]. We show here that immediately after irradiation γH2Av foci form inside the DAPI-rich regions but are rapidly removed to the periphery of this nuclear domain in agreement with recent data of Chiolo *et al*. [45]. This exclusion of foci from heterochromatic regions is likely due to the compact nature of heterochromatin, and consistent with the idea that heterochromatin is not well accessible to repair proteins. HP1a knockdown abrogated this migration, suggesting one of two things: First, HP1a depletion locally unwinds the chromatin and allows accessibility of heterochromatin to DDR proteins; hence there is no need for the foci to migrate closer to a euchromatic domain. Alternatively, the loss of HP1a may lead to the loss of additional components that are an integral part of heterochromatin, are dependent on HP1a for their localization and interact with DDR proteins. This interaction may be direct, through protein interactions, or indirect, through the regulation of gene expression by HP1a [19].

Since MU2 is also a part of heterochromatin and interacts with ATM (unpublished observations), it is possible that a decrease in the levels of MU2 would have additional effects on the recognition of heterochromatic breaks. We have shown previously that knockdown of MU2 by RNAi in S2 cells affects the kinetics of γH2Av foci formation [12]. MU2 binds to HP1 in untreated cells, but after irradiation MU2 accumulates at IRIFs, while the foci are removed from HP1-rich chromocenter. It is thus likely that MU2 and HP1 play very different roles in recognition of radiation induced DNA damage.

### HP1a affects cell cycle parameters

Drosophila HP1a is known to have pleiotropic affects, including the formation and maintenance of heterochromatin, telomere capping and transcription regulation [19]. Classical studies on position effect variegation have shown that HP1a is a negative regulator of transcription through the formation of heterochromatin. On the other hand, loss of HP1a in Kc cells causes a considerable decrease in the transcripts of genes involved in replication and mitosis [18]. HP1a knockdown in Kc cells further leads to an increase in apoptosis and a decrease in the number of cells in S and G2 phases of the cell cycle. In addition, a decrease in the level of HP1a produced a decrease in the transcripts of mitotic checkpoint genes *Bub1* and *Bub3* [18]. A decrease of PH3 in HP1a depleted cells leads to defects in metaphase and anaphase [46]. Using the S2 cell system to examine the effects of a decrease in HP1a, we could recapitulate the defects in cell cycle, however we did not observe an increase in the levels of apoptosis, which may be explained based on the aneuploid nature of S2 cells. Cells started showing pronounced defects after metaphase, suggesting that HP1a regulates the metaphase to anaphase transition, possibly by interacting with anaphase promoting complex/cyclosome (APC/C). We also observed that upon irradiation HP1a depleted cells did not arrest at the G2/M checkpoint. Our studies using wing imaginal discs showed that HP1a is involved in the response to DSBs, and also regulated the G2/M checkpoint. These observations clearly suggest that HP1a is involved in the progression of cells through mitosis and may mediate proper chromosome condensation.

### DNA double strand breaks during mitosis

The mechanisms of DNA damage response during interphase of the cell cycle have been characterized extensively, however little is known about the DDR response during mitosis, during which the basic features of chromosomes are inherently different from interphase chromatin. Phosphorylation of histone H3 at Ser 10 leads to chromosome condensation coupled with ejection of HP1a, -b and -c from chromatin [47]. Irradiation of cells in early prophase leads to cell cycle arrest, preventing the condensation of chromosomes or formation of astral spindle structures in the cytosol and reverting the cells to G2 phase. However, when the irradiation is in late prophase, after the G2/M checkpoint, the cells go through mitosis with DNA breaks [48]. It has been suggested that the there is a delay in the metaphase to anaphase transition in irradiated mitotic cells that is attributed to the activation of a spindle assembly checkpoint [49]. The repair of these breaks occurs only after the cell enters interphase and the foci disappear [50]. We observed that S2 cells mounted a response to irradiation in the different phases of mitosis. However, the γH2Av foci completely disappeared during telophase, suggesting that repair mechanisms may be activated as soon as anaphase is completed, chromatin starts to decondense and cells start organizing the nuclear membrane. Mitotic cells exhibited brighter γH2Av foci than interphase cells, suggesting a role of H3S10 phosphorylation or histone H1 phosphorylation in the phosphorylation of H2Av, possibly through the exclusion of HP1. We have shown that heterochromatin excludes γH2Av foci and decondensation alleviates this affect. Mitotic chromosomes are far more condensed than heterochromatic regions, but still do not exclude foci, suggesting that the nature of chromatin is decisive in the regulating the formation of γH2Av.

## Methods

### Drosophila strains

Drosophila stocks were maintained at 25° C on cornmeal, molasses medium with dry yeast added to the surface. The wild type Oregon R (OreR) strain was used as a control in all experiments, except where stated otherwise. *Su(var)205^05^/CyO*, *GFP* was a kind gift from Michael Brodsky (U Mass, Worcester). Double mutants of *Su(var)205^05^/CyO* and *mu2^a^* were generated by standard genetic crosses. HP1a RNAi fly lines V31994 and V31995 (with dsRNA constructs under a UAS promoter) were obtained from the Vienna Drosophila Resource Center, Vienna. To perform RNAi in wing imaginal discs, V31994 or V31995 females were crossed with *y w^67c23^*; *en-Gal4/en-Gal4* males and experiments were performed using third instar larvae.

### Cloning, cell culture, antibodies and transfection

Full length HP1a, -b and -c transcripts were amplified using RT (reverse transcription) PCR and were cloned into Drosophila expression vector pAGW (N-terminal eGFP Tag) using the Gateway cloning system (Invitrogen, Carlsbad). pAGW-MU2 has been described previously [12]. The region of MU2 that binds to HP1 was deleted and the remaining portion ligated and expressed in S2 cells. The full-length Drosophila ATM clone was a kind gift from Michael Brodsky. The region of MU2 that binds to HP1a (aa 900-1000) was amplified from a cDNA clone and inserted into a pGEX-4T vector for expression as a GST fusion protein. The expressed GST fusion protein was immobilized on Glutathione Sepharose (Amersham) and was used for further experiments. The sequences of the full length ORFs were confirmed before proceeding with further experiments. Exponentially growing S2 cells were seeded in 8 well-chambered slides and transfected with pAGW-dHP1a, -b, -c and dATM plasmid using Effectene (Qiagen). Mouse anti-PH3 antibody, clone 3H10 (Millipore, USA), and mouse anti-HP1, clone C1A9, (Developmental Studies Hybridoma Bank, Iowa) were used at a dilution of 1∶50. HP1b and -c antibodies were a kind gift from Joan Font-Burgada (Institute of Molecular Biology, Barcelona, Spain).

### Preparation of nuclear extracts and co-immunoprecipitation

Nuclear extracts were prepared from S2 cells using the protocol of Dignam *et al.* [51]. Co-IP experiments were performed as described [12]. Briefly, embryos were collected for 6 h time intervals and were lysed in buffer containing 10 mM Tris, pH 8.0, 200 mM NaCl, 1% NP40, 0.1 mM EDTA, 0.1 mM EDTA, and protease inhibitors. Protein concentrations were estimated, and extracts were stored at −70°C. 500 µg of the protein from the nuclear extract were used to bind equal amounts of anti-GFP antibodies conjugated to agarose beads (Santa Cruz Biotech). After washing the bound proteins were detected by Western blot analysis.

### HP1a RNAi in S2 cells

RNAi was performed in S2 cells according to established protocols [52], [53]. Full length ORFs of HP1a were amplified from the cDNA clone using primers with T7 promoter binding sites at the 5′ and the 3′ ends. As a negative control, we PCR amplified a 750 bp sequence from the bacterial cloning plasmid using the same strategy. The PCR products were gel purified, an *in vitro* transcription reaction was performed, and the dsRNA was purified using Megascript T7 kit (Ambion Inc.) according to the manufacturers instructions. S2 cells were grown as described previously, plated at a density of 1×10^5^ in 6 well plates, and 15 µg of dsRNA was added to each well in serum free medium. Cells were incubated with dsRNA for 45 minutes and equal mounts of mM3 BPYE medium containing 20% FCS was added. After 3 days of incubation with dsRNA, Western blots were performed to detect the levels of HP1a, -b and -c.

### Immunofluorescence

Immunostaining of S2 cells was performed as described [12]. Cells were incubated with primary rabbit anti-γH2Av at 1∶1000 dilution (Rockland Biochemicals, MD) and with mouse anti-HP1a antibodies (1∶100), rabbit anti-HP1b and -HP1c antibodies (1∶500), and mouse anti-PH3 (1∶500). For staining mitotic cells, the cells were permeabilized with PBS containing 0.3% Triton ×100 and stained with a monoclonal mouse anti-PH3 antibodies [48]. Slides were mounted in SlowFade Gold antifade reagent (Invitrogen) and were visualized using confocal microscopy. Fluorescence intensity was measured in PH3-positive cells using Image J software.

### HP1a RNAi, apoptosis detection and immunostaining of wing imaginal discs

HP1a RNAi lines, V31994 and V31995, were crossed to tissue-specific GAL4 stocks with expression specific to wing imaginal discs or oocytes. Wing imaginal discs were dissected from the controls and GAL4/UAS dsRNAi expressing flies and stained to assess the effects of HP1a depletion on apoptosis and G2/M arrest. Detection of apoptosis in wing imaginal discs was performed by staining with acridine orange as described previously [38], [54]. To study the effects of HP1a knockdown on cell cycle arrest, wandering third instar larvae were irradiated with 10 Gy of IR at a dose rate of 5 Gy/min using a Cs source. Wing imaginal discs were dissected three hours after IR and fixed in 4% paraformaldehyde as described previously [55]. Discs were washed in buffer (PBS with 0.1% Triton ×100) and block-permeabilized for 20 minutes in PBS containing 1% BSA and 0.3% Triton ×100. Wing discs were incubated with primary antibodies to mouse PH3 (1∶1000) and rabbit anti-γH2Av (1∶500) (Rockland Biochemicals). Slides were mounted in SlowFade Gold antifade reagent (Invitrogen) and were visualized using confocal microscopy. The wing discs were flattened and numbers of apoptotic and mitotic cells were counted from at least five discs in three independent experiments, as described earlier [56].

### RT-PCR analysis of HP1a

RNA samples were made using RNasy mini kit (Qiagen) according to the manufacturer's instructions and reverse transcribed using oligo(dT) and the SuperScript first-strand synthesis system for RT PCR (Invitrogen). Levels of HP1a were measured using RT PCR.

### Laser micro-irradiation and live cell imaging

Live cell imaging combined with laser micro-irradiation was carried out as described previously [57], [58], [59]. Exponentially growing S2 cells were transfected with pAGW-dHP1a, -b, -c or pAGW-ATM, or -H2Av plasmid using Effectene (Qiagen). 24 h prior to exposure cells were sensitized with 10 µM BrdU. Fluorescence in living cells was monitored by using an Axiovert 200M microscope (Carl Zeiss MicroImaging, Thornwood, NY, USA). A 364-nm continuous wave laser (Spectra-Physics, Mountain View, CA, USA) was directly coupled to the epifluorescence path of the microscope. DSBs were generated in a defined area of the nucleus by micro-irradiation with the 364-nm laser. All measurements were corrected for nonspecific bleaching during monitoring, and the experiments were performed in triplicate. Cells were sensitized with 10 µM BrdU 24 h prior to exposure to laser. The experiments were performed three times with more than 10 cells each time.

### FACS analysis of S2 cells

Control, irradiated (10 Gy at a dose rate of 5 Gy/min) or HU treated (10 mM for 16 h) S2 cells were fixed in 70% ethanol and FACS analysis of PI stained nuclei was performed as described [18]. Fluorescence was measured using a BD FACSort, and cell cycle stages were analyzed using the Modfit software. To detect changes in the levels of histone modifications, S2 cells were treated with 10 Gy of irradiation or with dsRNA for HP1a. Cells were fixed at 4°C for 30 minutes using 1.5% formaldehyde followed by a second fixation using 70% methanol. Fixed S2 cells were washed with staining solution (PBS containing 1% BSA) and permeabilized using PBST (PBS containing 0.1% Triton ×100) for 10 minutes at room temperature. Cells were blocked with PBS containing 10% normal goat serum for 30 minutes at room temperature. Cells were stained with mouse anti-HP1a, rabbit anti-γH2Av (1∶500), rabbit anti-H3K9Me3 and monoclonal anti-PH3 antibodies. After one wash with staining solution, cells were stained with secondary antibodies, goat anti-rabbit Alexafluor 488 or goat anti-mouse Alexfluor 647. 10,000-gated events were acquired and analyzed using cell quest software. Fluorescence was measured using a BD LSR II (Becton Dickinson), and data were analyzed using Cellquest software.

### Immunostaining of *Drosophila* embryos

Wild-type (OreR) and mutant (*mu2^a^*) embryos were collected on grape juice agar plates for 2 h at 25° C. Collected embryos were washed in embryo wash buffer (PBS containing 0.3% Triton ×100) and dechorionated in 50% Clorox for 2 min. Embryos were transferred to a 15-ml screw-top tube containing 4 ml of fix buffer. Then 5 ml of heptane and 1 ml of 37% formaldehyde were added to the fix buffer and shaken by inversion for 20–25 min. The embryos were de-vitillenized by adding 100% methanol in heptane. Embryos were blocked in PBST containing 10% NGS and stained with primary antibodies to HP1a and detected using goat anti-rabbit AlexaFluor 594 and visualized under the confocal microscope [60].

## Supporting Information

Figure S1 **Y2H analysis of HP1a and MU2 interactions.** MU2 protein fragments, shown in the lower table, were expressed as bait, and Y2H experiments were performed by screening these fragments against S2 and embryonic libraries as described [25]. The number of hits represent the times that fragment interacted with HP1a. No interactions with other prey fragments were seen. The table shows the details of bait (MU2) and prey (HP1a) fragments and the library used to screen the interaction. All of the prey fragments identified carried the HP1a chromoshadow domain.
(TIF)

Figure S2 **HP1 and MU2 during the cycle 14 stage of embryogenesis.** Females of the genotype *y w*; *P{mGFP-MU2}/CyO* were allowed to lay eggs on grape juice agar plates. The eggs were dechorionated, stained with DAPI and anti-HP1 (red), and observed under a confocal microscope. The top row shows the OreR control. The middle row shows *mu2^a^* embryos. While there is no change in the localization of HP1 in nuclei that have migrated to the surface, many nuclei are slow to migrate. The third row shows staining of OreR embryos with mGFP-MU2 (green) and DAPI. It can be observed that MU2 is primarily cytoplasmic and the DAPI rich regions are apical, suggesting the establishment of heterochromatin.
(TIF)

Figure S3 **HP1b and HP1c are not localized to IRIF.** Irradiated S2 cells are shown labeled with γH2Av (green), DNA as identified by DAPI, and either HP1b (top row) or HP1c (bottom row) in red. The HP1b and HP1c staining pattern in irradiated cells does not change in comparison to controls; neither HP1b nor HP1c co-localize with the γH2Av foci upon irradiation as shown in the merged images.
(TIF)

Figure S4 **Real time localization of eGFP-tagged H2Av to laser-induced DSBs.** (A) S2 cells were grown in 8-well chambered slides and transfected with pAGW-H2Av (eGFP-tagged H2Av expressed under the control of the actin 5C promoter) using standard procedures. Cells were sensitized to laser-induced DSBs using 10 µM BrdU for 16 h. Regions of interest were drawn over the cell nucleus using Carl Zeiss software and cells were exposed to 360 nm continuous wave UV laser and monitored over time. Localization was visible as a fluorescence streak at the region of interest. (B) Graphical representation of the increase in the fluorescence intensity at the region of interest.
(TIF)
