# Supplementary figures and images for: MU2 and HP1a Regulate the Recognition of Double Strand Breaks in Drosophila melanogaster
Source: PLoS One. 2011 Sep 23;6(9):e25439. doi: 10.1371/journal.pone.0025439 (PMC3179522; doi:10.1371/journal.pone.0025439)

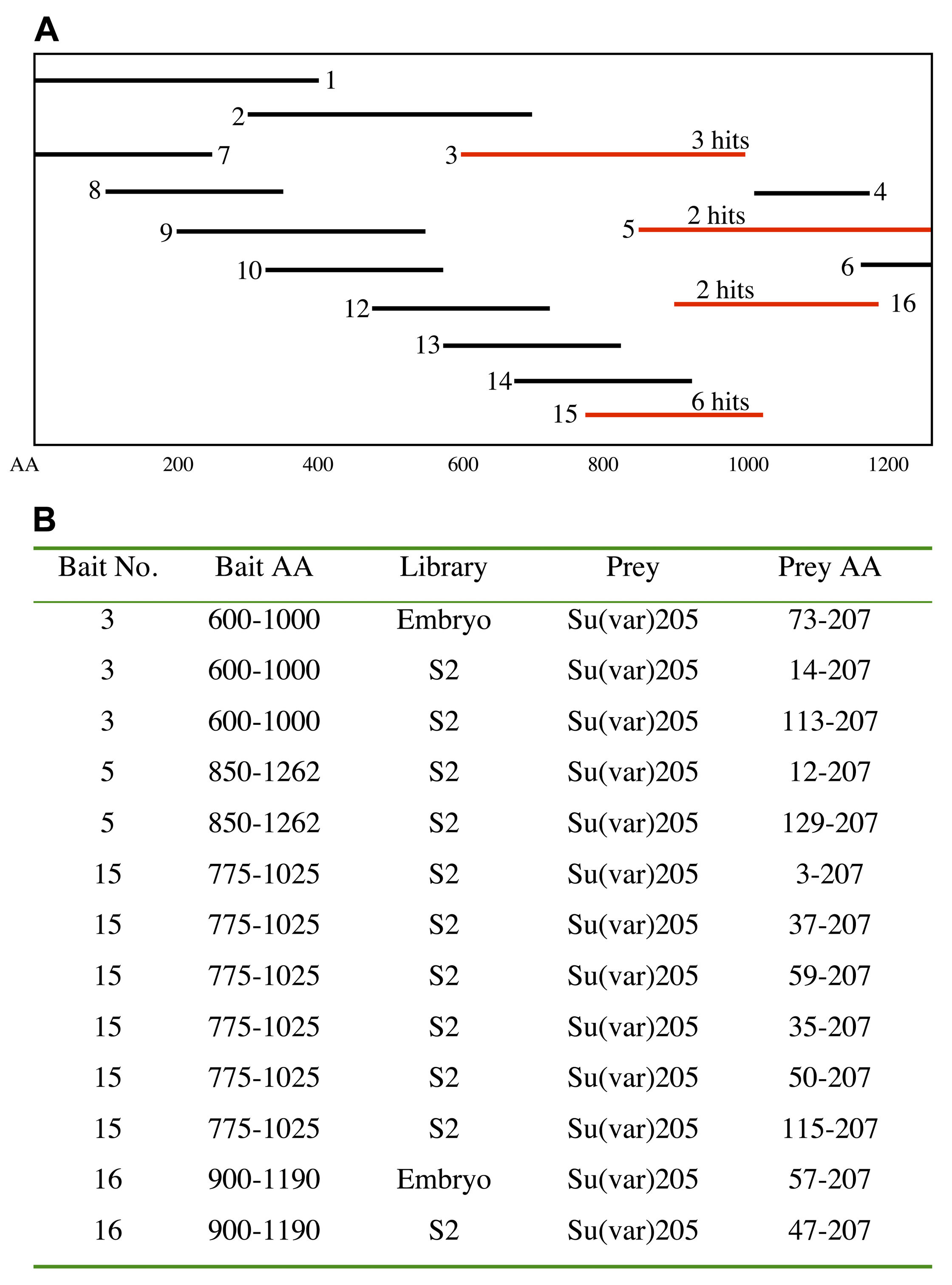

Supplement: Figure S1 — Y2H analysis of HP1a and MU2 interactions. MU2 protein fragments, shown in the lower table, were expressed as bait, and Y2H experiments were performed by screening these fragments against S2 and embryonic libraries as described [25]. The number of hits represent the times that fragment interacted with HP1a. No interactions with other prey fragments were seen. The table shows the details of bait (MU2) and prey (HP1a) fragments and the library used to screen the interaction. All of the prey fragments identified carried the HP1a chromoshadow domain. (TIF) [file pone.0025439.s001.tif]

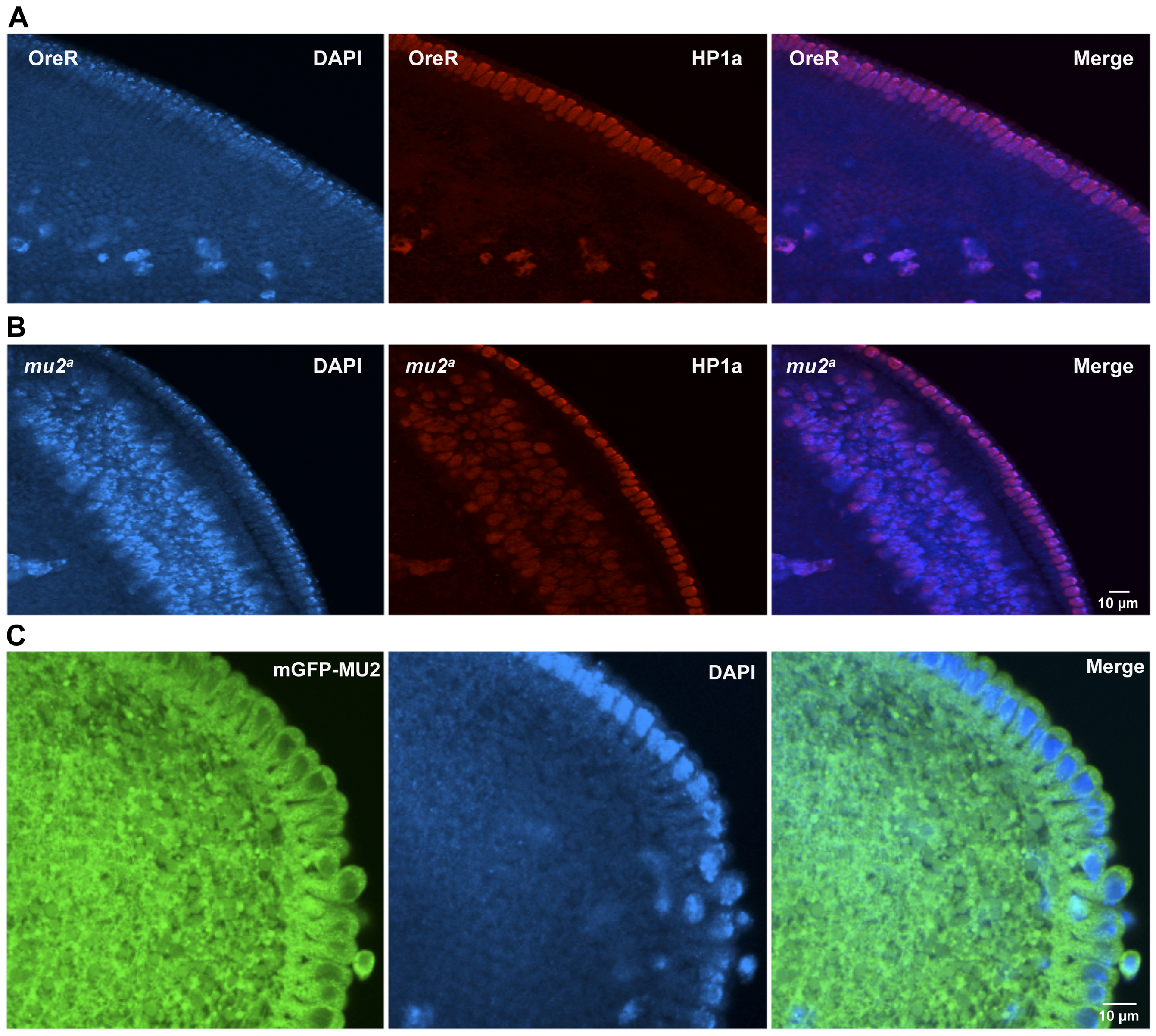

Supplement: Figure S2 — HP1 and MU2 during the cycle 14 stage of embryogenesis. Females of the genotype y w; P{mGFP-MU2}/CyO were allowed to lay eggs on grape juice agar plates. The eggs were dechorionated, stained with DAPI and anti-HP1 (red), and observed under a confocal microscope. The top row shows the OreR control. The middle row shows mu2a embryos. While there is no change in the localization of HP1 in nuclei that have migrated to the surface, many nuclei are slow to migrate. The third row shows staining of OreR embryos with mGFP-MU2 (green) and DAPI. It can be observed that MU2 is primarily cytoplasmic and the DAPI rich regions are apical, suggesting the establishment of heterochromatin. (TIF) [file pone.0025439.s002.tif]

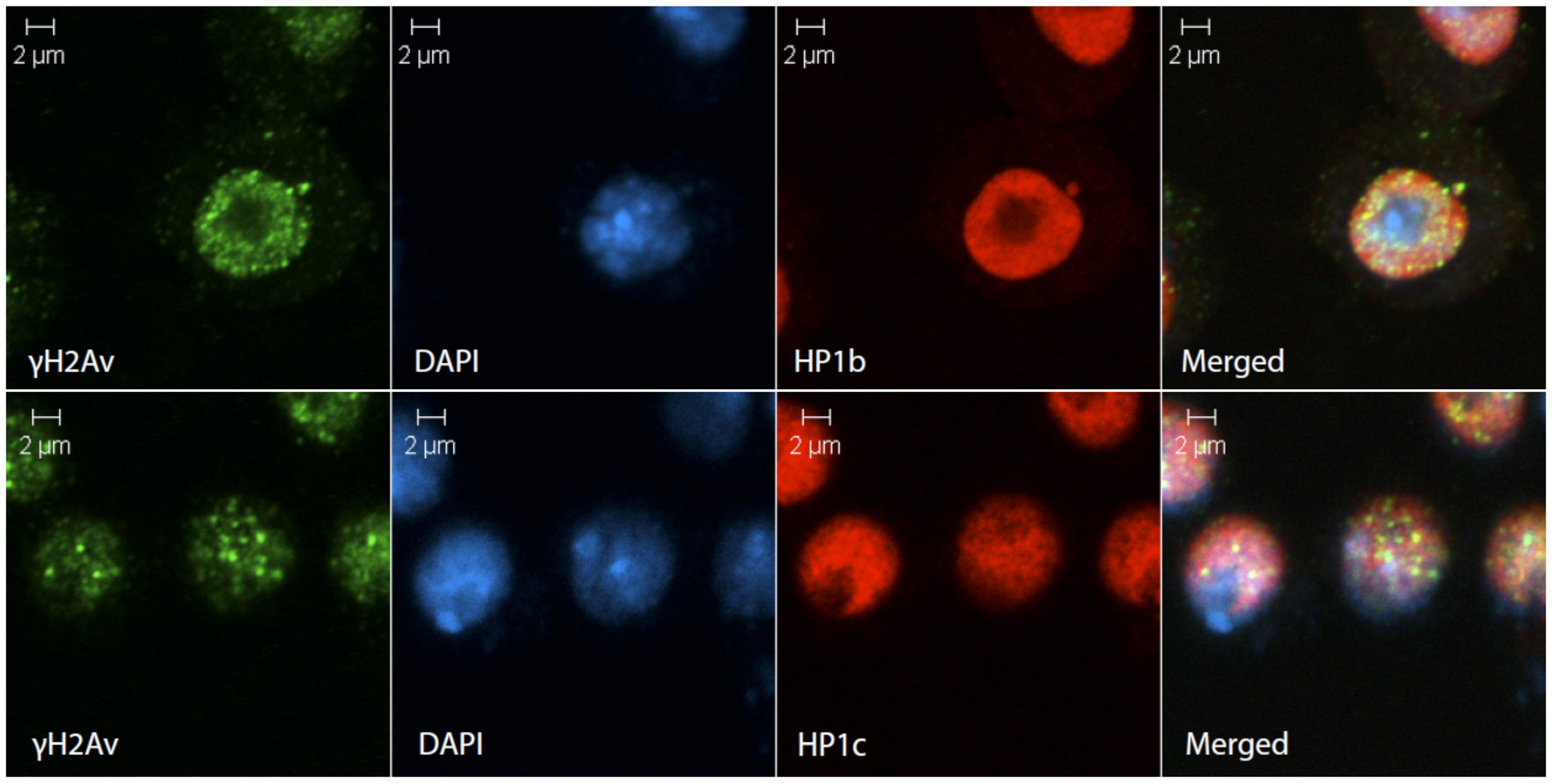

Supplement: Figure S3 — HP1b and HP1c are not localized to IRIF. Irradiated S2 cells are shown labeled with γH2Av (green), DNA as identified by DAPI, and either HP1b (top row) or HP1c (bottom row) in red. The HP1b and HP1c staining pattern in irradiated cells does not change in comparison to controls; neither HP1b nor HP1c co-localize with the γH2Av foci upon irradiation as shown in the merged images. (TIF) [file pone.0025439.s003.tif]

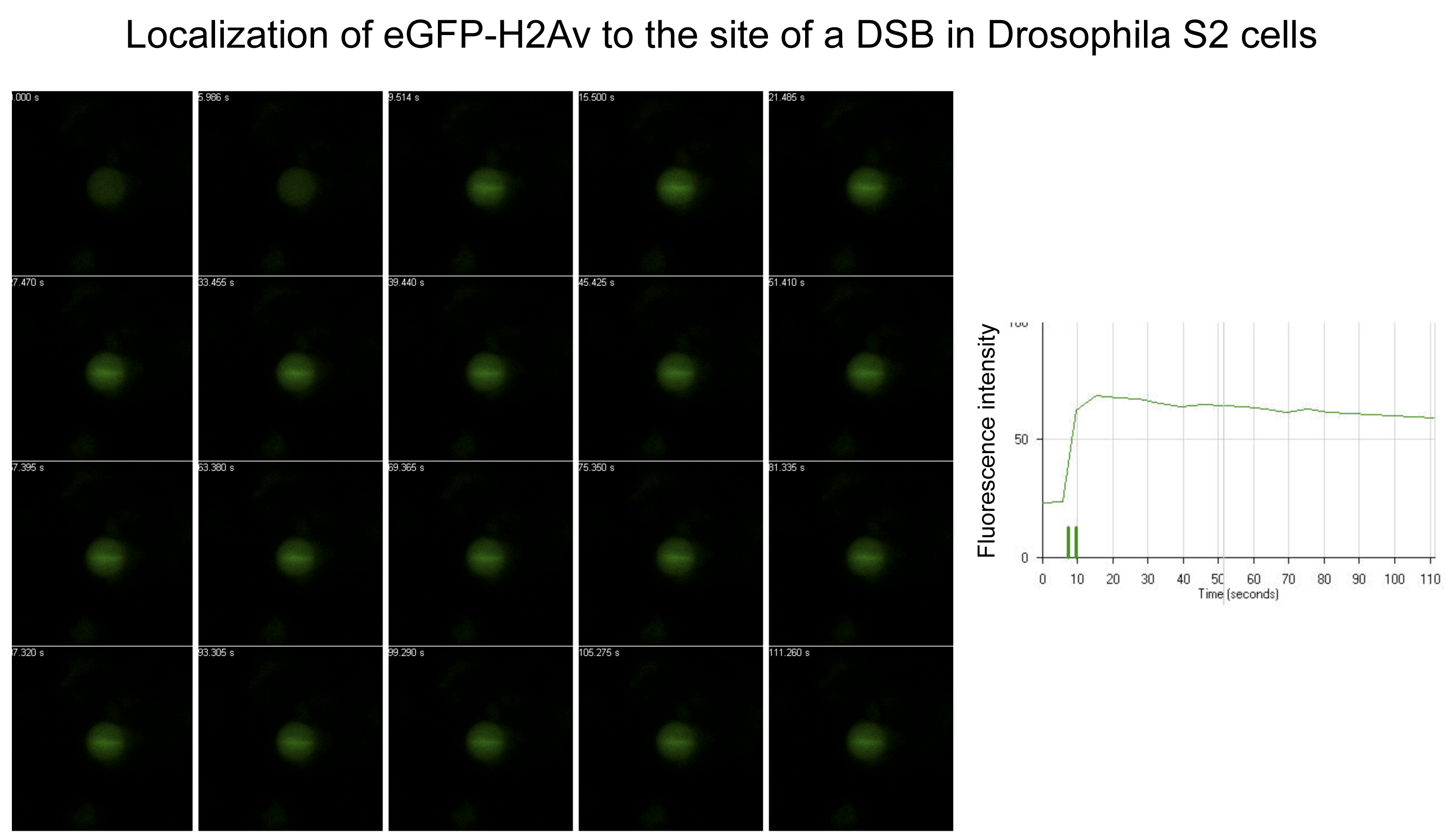

Supplement: Figure S4 — Real time localization of eGFP-tagged H2Av to laser-induced DSBs. (A) S2 cells were grown in 8-well chambered slides and transfected with pAGW-H2Av (eGFP-tagged H2Av expressed under the control of the actin 5C promoter) using standard procedures. Cells were sensitized to laser-induced DSBs using 10 µM BrdU for 16 h. Regions of interest were drawn over the cell nucleus using Carl Zeiss software and cells were exposed to 360 nm continuous wave UV laser and monitored over time. Localization was visible as a fluorescence streak at the region of interest. (B) Graphical representation of the increase in the fluorescence intensity at the region of interest. (TIF) [file pone.0025439.s004.tif]
